# Catastrophic health care expenditure due to septic shock and dengue shock in Vietnam

**DOI:** 10.1093/trstmh/trz064

**Published:** 2019-07-24

**Authors:** Angela McBride, B Thuy Duong, V Vinh Chau Nguyen, C Louise Thwaites, Hugo C Turner, V Hao Nguyen

**Affiliations:** 1 Department of Global Health and Infection, Brighton and Sussex Medical School, Falmer, Brighton, England; 2 Oxford University Clinical Research Unit, 764 Vo Van Kiet, Quan 5, Ho Chi Minh City, Vietnam; 3 Hospital for Tropical Diseases, 764 Vo Van Kiet, Quan 5, Ho Chi Minh City, Vietnam

**Keywords:** cost of illness, septic, severe dengue, shock

## Abstract

**Background:**

The cost of treatment for infectious shock in intensive care in Vietnam is unknown.

**Methods:**

We prospectively investigated hospital bills for adults treated for septic and dengue shock in Vietnam and calculated the proportion who faced catastrophic health care expenditures.

**Results:**

The median hospital bills were US$617 for septic shock (n=100) and US$57 for dengue shock (n=88). Catastrophic payments were incurred by 47% (47/100) and 13% (11/88) of patients with septic shock and dengue shock, respectively, and 56% (25/45) and 84% (5/6) fatal cases of septic shock and dengue shock respectively.

**Conclusions:**

Further advocacy is required to moderate insurance co-payments for costly critical care interventions.

## Background

In low- and middle-income countries (LMICs), where a high burden of critical illness is attributable to severe infection, intensive care units (ICUs) are rapidly expanding in number and capability. Although the cost-effectiveness of critical care for severe infection is understudied in LMICs, extrapolation of data from high-income settings suggests that the costly but lifesaving interventions provided in the ICU likely result in net economic gains to society.^1^ But despite the roll-out of universal health insurance in Vietnam, patients continue to contribute a significant proportion of their own hospital bills.^2^ Therefore the conversation around cost-effectiveness of critical care in LMICs should be tempered with an understanding of the burden that such out-of-pocket payments place on patients and their families.

We investigated the total hospital bills and insurance contributions for adults treated for the two most frequent causes of infectious shock in southern Vietnam, septic shock and dengue shock, and the proportion of patients/families who faced catastrophic payments for health care.

## Methods

We conducted this prospective observational study between November 2014 and January 2016 on adult ICU at the Hospital for Tropical Diseases, a tertiary referral hospital for infectious diseases in Ho Chi Minh City, Vietnam. After obtaining written consent, we recorded patient demographics, clinical severity, hospital mortality, hospital bill, insurance status and contributions for adults (≥15 y) admitted with septic shock and dengue shock (with or without haemorrhagic complications or multi-organ failure) as defined by Sepsis 3^3^ and the World Health Organization criteria,^4^ respectively. In line with Sustainable Development Goal 3.8.2, a catastrophic payment was defined as occurring when the patient’s out-of-pocket health care payment was >10% of the average annual household expenditure in Vietnam,^5^ which was estimated to be US$2986.51 in 2014.^2,6^ Cost data were converted to US$ using the average exchange rate over the study period.^6^ Statistical analyses were conducted using Stata version 8.0 (StataCorp, College Station, TX, USA); hospital bills were compared by Wilcoxon rank-sum test.

## Results

A total of 100 patients were treated for septic shock and 88 had dengue shock. The median hospital bill for septic shock (US$617 [interquartile range {IQR} US$283–1170, range US$56–9144]) were >10-fold higher than for dengue shock (US$57 [IQR US$37–92, range US$10–10 379]). Patients admitted with septic shock were older, had more comorbidities and had higher Acute Physiology, Age, Chronic Health Evaluation (APACHE) II scores at baseline than patients with dengue shock. Requirement for vasopressor support, mechanical ventilation, haemofiltration and transfusion was more common among patients with septic shock than dengue shock (Table 1).

**Table 1.** Demographics, hospital bills and insurance contributions for patients with septic shock and dengue shock

	Septic shock (n=100)	Dengue shock (n=88)
Patient characteristics		
Age, years, median (25th–75th centile)	53 (41–63)	25 (19–32)
Male, n (%)	55 (55)	31 (35)
Charlson Comorbidity Index, median (25th–75th centile)	1 (0–3)	0 (0–0)
Duration of ICU admission, days, median (25th–75th centile)	3 (2–5)	1 (1–2)
Duration of hospital admission, days, median (25th–75th centile)	7 (2–14)	5 (4–7)
Death in hospital, n (%)	45 (45)	6 (6.8)
Baseline APACHE II score, median (25th–75th centile)	18.5 (14–23)	4 (3–8)
Organ support required, n (%)		
Vasopressors	86 (86)	12 (13.6)
Mechanical ventilation	51 (51)	11 (12.5)
Haemofiltration	27 (27)	9 (10.2)
Transfusion (packed red cells, fresh frozen plasma, platelets)	22 (22)	11 (12.5)
Hospital bill and catastrophic costs		
Proportion with health insurance (any), n (%)	52 (52)	32 (36.4)
Hospital bill, US$, median (25th–75th centile)	617 (283–1170)	57 (37–92)
Proportion of hospital bill co-paid by insured patients, %, median (25th–75th centile)	26.3 (23.4–36.1)	22.4 (21.9–23.8)
Proportion with catastrophic expenses, n (%)	47 (47.0)	11 (12.5)
Proportion of patients with insurance with catastrophic expenses, n (%)	14/47 (29.8)	4/11 (36.4)
Costs of health care in non-survivors	Septic shock (n=45)	Dengue shock (n=6)
Proportion of bereaved families with catastrophic expenses, n (%)	25 (55.5)	5 (83.3)
Hospital bill, US$ (patients with insurance), median (25th–75th centile)	241 (95–960)	521 (236–1321)
Hospital bill, US$ (patients without insurance), median (25th–75th centile)	960 (301–1468)	7773 (5624–7956)

Cost data are expressed in nominal prices.

The proportion of patients incurring catastrophic payments was 47% (47/100) for septic shock and 13% (11/88) for dengue shock. The families of 56% (25/45) and 83% (5/6) of patients who died from septic shock and dengue shock, respectively, incurred catastrophic expenses. Hospital bills were significantly greater for patients who died from vs survived septic shock (p<0.001) and dengue shock (p<0.001).

The proportion of patients with health insurance was 52% (52/100) and 36% (32/88) for septic shock and dengue shock, respectively. Hospital bills were significantly higher among uninsured vs insured patients with septic shock (p<0.001) and dengue shock (p<0.001). Among patients with catastrophic expenditures, 30% (14/47) and 36% (4/11) of patients with septic shock and dengue shock, respectively, had health insurance.

## Discussion

This study highlights the frequent requirement for patients and families to make catastrophic payments for treatment of infectious shock in Vietnam. This is especially true for families of non-survivors, who may face the double blow of losing an economically active member of their household and having to pay a large health care bill. The median cost of treatment for septic shock was much greater than for dengue shock. This is likely due to a combination of a longer ICU stay, increased requirements for organ support and the cost of antibiotic therapy. Patients with septic shock more frequently had known comorbidities when compared with those with dengue shock. This may also have contributed to the increased length of ICU stay and organ support requirements.

Having health insurance protected against but did not completely prevent catastrophic hospital bills. Thus, together with the continued expansion of health insurance coverage in Vietnam, strategies are needed to protect insured patients from very large co-payments for the high-cost interventions they receive in the ICU.

Very few studies have reported patient costs for critical care in LMICs. A single-centre study in Thailand (an upper-middle income country) reported the median hospital bill for patients with septic shock (2011–2015) as US$2792. The authors of the study did not find a significant difference in costs between survivors and non-survivors, which is at odds with our results.^7^

Our study has limitations. We did not have access to itemized bills and our data were collected from a tertiary hospital, thus our patients may have had more severe disease and/or access to high-cost medical interventions not available in district or provincial hospitals. We did not account for health care costs before or after hospitalization. In addition, we did not calculate patients’/informal caregivers’ direct non-medical and productivity costs; a longitudinal study is under way to investigate these. Including such costs will likely increase the proportion facing catastrophic health expenditures. Further, applying an arbitrary threshold for catastrophic expenditures may not reflect the true ability of patients and families to absorb large health care bills.

## Conclusions

In Vietnam, both insured and uninsured patients treated in the ICU for septic shock and dengue shock frequently face catastrophic bills for their hospital care. Our results indicate the need for ongoing advocacy with policymakers to moderate co-payments for high-cost critical care interventions.
